# Fill Patterns of Glucose-Lowering Drugs with Cardiovascular and Kidney Benefits in the Rural and Urban United States, 2012–2021

**DOI:** 10.1007/s11606-025-09784-0

**Published:** 2025-08-04

**Authors:** Kyle Steiger, Kavya Sindhu Swarna, Jeph Herrin, Rozalina G. McCoy

**Affiliations:** 1 Internal Medicine Residency, Mayo Clinic, Rochester, MN USA; 2 Robert D. and Patricia E. Kern Center for the Science of Health Care Delivery, Mayo Clinic, Rochester, MN USA; 3 OptumLabs, Eden Prairie, MN USA; 4 Section of Cardiovascular Medicine, Yale School of Medicine, New Haven, CT USA; 5 Division of Endocrinology, Diabetes, and Nutrition, Department of Medicine, University of Maryland School of Medicine, 670 West Baltimore Street, Baltimore, MD 21201 USA; 6 University of Maryland Institute for Health Computing, North Bethesda, MD USA

**Keywords:** Sodium/glucose cotransporter-2, Glucagon-like peptide, Heart failure, Chronic kidney disease, Cardiovascular disease

## Abstract

**Background:**

Glucagon-like peptide-1 receptor agonists (GLP1RA) and sodium-glucose cotransporter-2 inhibitors (SGLT2i) improve cardiovascular and kidney outcomes, but accessibility concerns persist. Whether access is worse in rural communities is unknown.

**Objective:**

To examine fills of GLP1RA/SGLT2i by adults with type 2 diabetes in rural and urban US counties.

**Design:**

Cross-sectional use claims from OptumLabs® Data Warehouse, 2012–2021.

**Patients:**

Adults with type 2 diabetes.

**Main Measures:**

We examined rates and odds of fills of GLP1RA or SGLT2i, adjusting for time period, age, sex, presence of cardiorenal comorbidities (heart failure [HF], atherosclerotic cardiovascular disease [ASCVD], chronic kidney disease stages 3–4 [CKD]), and rurality.

**Key Results:**

The study included 2,579,577 adults with type 2 diabetes (mean age 63.9; 49.6% female). Compared to 2012–2015, fills of GLP1RA/SGLT2i increased in 2016–2018 (OR 2.49; 95% CI 2.47–2.52) and 2019–2021 (OR 4.35; 95% CI 4.30–4.39). By 2019–2021, the percentages of patients filling GLP1RA/SGLT2i in cities, small towns, and remote areas were 18.8%, 18.8%, and 17.4%, respectively. Small town residents had higher odds of fills than those in cities (OR 1.05; 95% CI 1.04–1.06). Older age was associated with lower fill rates; compared to 18–44 year olds, odds for those 45–64 years were OR 0.88 (95% CI 0.87–0.89), 65–74 years: OR 0.48 (95% CI 0.47–0.48), and ≥ 75 years: OR 0.17 (95% CI 0.17–0.18). Men were less likely to fill GLP1RA/SGLT2i than women (OR 0.91; 95% CI 0.90–0.92). Presence of ASCVD and CKD were associated with greater odds of filling GLP1RA/SGLT2i (OR 1.03 [95% CI 1.01–1.05] and OR 1.12 [95% CI 1.10–1.14], respectively), while HF was associated with lower odds (OR 0.86 [95% CI 0.85–0.88]).

**Conclusions:**

GLP1RA and SGLT2i fills increased over time but remained low overall. They were filled more often by younger people, women, those with ASCVD or CKD (but not with HF), and in small towns.

**Supplementary Information:**

The online version contains supplementary material available at 10.1007/s11606-025-09784-0.

## INTRODUCTION

The robust evidence on the cardiovascular and kidney benefits of glucagon-like peptide-1 receptor agonists (GLP1RA) and sodium-glucose cotransporter-2 inhibitors (SGLT2i) in adults with type 2 diabetes with established atherosclerotic cardiovascular disease (ASCVD), heart failure (HF), and chronic kidney disease (CKD) and in those at high risk for these conditions has shifted the standard of type 2 diabetes care to prioritize the use of these drug classes for both glycemic management and ASCVD, HF, and CKD risk reduction.^1^ Despite evidence and guidelines,^1^ it can take decades for care adjustments to be adopted^2^ with greater gaps among underserved communities.^3^ Prior studies have revealed slow adoption of these diabetes medications among Black individuals, women, those with low income, those living in socioeconomically deprived areas, and those with Medicare Advantage as opposed to private health plans,^4–8^ making it essential to monitor for and address disparities in the adoption of evidence-based care.

People living in rural areas of the United States—including small towns and remote areas—have a higher prevalence of diabetes, a greater risk of developing diabetes-related complications,^9^ and higher rates of mortality from diabetes, heart disease, and kidney disease than those living in urban areas.^10^ Some of these gaps may be driven by limited access to health care in rural compared to urban areas,^11–13^ but whether rural communities experience gaps in evidence-based type 2 diabetes management has not been examined.

Therefore, we sought to assess the utilization of medications with cardiovascular and kidney benefits among people with type 2 diabetes in rural and urban areas of the United States, additionally seeking to assess the impacts of age, sex, and cardiovascular and kidney comorbidities on GLP1RA and SGLT2i use over time.

## MATERIALS AND METHODS

### Data Source and Study Design

This cross-sectional study used medical and pharmacy claims data of enrollees in commercial and Medicare Advantage health plans included in OptumLabs® Data Warehouse (OLDW), a de-identified administrative claims database composed of health plans offered by a large nationwide insurance company.^14^ It contains longitudinal health information for over 130 million enrollees, representing a diverse mixture of ages, racial and ethnic groups, income levels, and geographic regions across the USA with slight overrepresentation of Midwestern and Southern populations. As all data were accessed after statistical de-identification, the study was exempt by Mayo Clinic from Institutional Review Board review.^15^ Results are reported in accordance with STROBE guidelines for cross-sectional studies.^16^

### Study Population

We used Healthcare Effectiveness Data and Information Set (HEDIS) criteria^17^—either one acute care diabetes diagnosis code, two nonacute care diabetes E&M codes, or a fill for a glucose-lowering medication other than metformin—to identify individuals with diabetes included in OLDW between January 1, 2011 and December 31, 2020. We excluded those without geographic data or 12 months of follow-up and those with type 1 diabetes, end-stage kidney disease, and minors (aged < 18 years) (Table S1; Figures S1-S2).

### Independent Variables

Patient age, sex, and 5-digit ZIP code were ascertained from OLDW enrollment files. ZIP codes were used to determine rural status, defined using Rural–Urban Commuting Area (RUCA) codes and categorized as cities (RUCA codes 1–3: metropolitan or micropolitan areas with population > 50,000 people), small town rural (RUCA codes 4–9: population 2500–50,000 people), and remote rural (RUCA 10: population < 2500 people), as previously described.^9,18–20^ Baseline comorbidities were ascertained during the 12 months following the HEDIS date (Table S2). These were then used to categorize the study population by comorbidities: (1) those without ASCVD, HF, or CKD; (2) those with ASCVD (cerebrovascular disease, coronary artery disease, and peripheral arterial disease); (3) those with HF; and (4) those with CKD. Comorbidities were established using International Classification of Diseases (ICD) 9 and 10 code sets (Table S3). These groupings were not mutually exclusive, as patients with more than one chronic condition of interest were included in all relevant groups.

### Outcomes

The 12-month period following HEDIS date was used to identify fills for GLP1RA and SGLT2i (Table S4). Any use of either a GLP1RA or SGLT2i was considered a binary positive outcome due to robust evidence for the benefit of either medication class among individuals with ASCVD, HF, and CKD.^1^

### Statistical Analyses

For each comorbidity grouping, we summarized patient-level covariates by geographic group, reporting frequencies and percentages for categorical variables and means and standard deviations for continuous variables. Differences between geographic groups were assessed using the Kruskal–Wallis test for continuous variables and a chi-square test for categorical variables. We then generated a series of logistic regression models comparing any fill of a GLP1RA or SGLT2i according to time period, age, sex, rurality, and cardiovascular and kidney comorbidity. Each model was then subset into each of the following time periods based on the year of final follow-up: 2012–2015, 2016–2018, and 2019–2021, as clinical data emerged supporting the use of SGLT2i and GLP1RA for cardiovascular and kidney risk reduction in late 2015 and early 2016.^21,22^ And the American Diabetes Association started recommending these medications in high-risk groups in 2019.^23^ Individual models were adjusted for time period, age, sex, rurality, and cardiovascular and kidney comorbidity as appropriate. Insurance type (commercial versus Medicare Advantage) was not included as a covariate in primary analyses because it has a strong effect on the use of pharmacotherapies and is therefore in the causal pathway for filling GLP1RA or SGLT2i agents. Insurance type is also collinear with age, which was included in the analysis. However, we conducted a sensitivity analysis including insurance type as a covariate to better delineate how much insurance type impacts receipt of evidence-based therapies. A two-sided *P*-value < 0.05 was considered statistically significant. All analyses were performed in SAS (version 9.4).

## RESULTS

This study included 2,579,577 adults with type 2 diabetes, of whom 256,602 (9.9%) had CKD, 945,515 (36.7%) had ASCVD, and 281,886 (10.9%) had HF, while 1,492,031 (57.8%) had none of these comorbidities (Table 1). Overall, 67,545 (2.6%) lived in remote areas, 370,626 (14.4%) lived in small towns, and 2,141,406 (83%) lived in cities. In most instances, baseline comorbidities were slightly more prevalent in small towns, while those in remote areas were slightly older and more frequently male. The extent of overlap between the comorbidity groups (i.e., cardiovascular and kidney multimorbidity) is provided in Table S5, and characteristics of each comorbidity grouping subset are provided in Tables S6-S9.

**Table 1:** Baseline Characteristics of the Study Sample, Stratified by Rurality. Data Are Summarized as Count and Percentage, Unless Otherwise Specified. Abbreviations: *CKD*, chronic kidney disease; *ASCVD*, atherosclerotic cardiovascular disease; *HF*, heart failure

	**Remote** (*N* = 67,545)	**Small town** (*N* = 370,626)	**City** (*N* = 2,141,406)	**Total** (*N* = 2,579,577)	***p* value**
Age, mean (SD)	65.4 (11.6)	64.7 (11.9)	63.7 (12.5)	63.9 (12.4)	< 0.0001
Age groups, years					< 0.0001
18–44	3540 (5.2%)	23,359 (6.3%)	171,569 (8.0%)	198,468 (7.7%)	
45–64	24,553 (36.4%)	138,955 (37.5%)	823,670 (38.5%)	987,178 (38.3%)	
65–74	24,211 (35.8%)	129,701 (35.0%)	708,472 (33.1%)	862,384 (33.4%)	
≥ 75	15,241 (22.6%)	78,611 (21.2%)	437,695 (20.4%)	531,547 (20.6%)	
**Sex**					< 0.0001
Female	32,347 (47.9%)	188,481 (50.9%)	1,058,892 (49.4%)	1,279,720 (49.6%)	
Male	35,198 (52.1%)	182,145 (49.1%)	1,082,514 (50.6%)	1,299,857 (50.4%)	
Baseline comorbidity status					
ASCVD	24,903 (36.9%)	141,626 (38.2%)	778,986 (36.4%)	945,515 (36.7%)	< 0.0001
CKD	6769 (10.0%)	38,869 (10.5%)	210,964 (9.9%)	256,602 (9.9%)	< 0.0001
HF	8275 (12.3%)	46,695 (12.6%)	226,916 (10.6%)	281,886 (10.9%)	< 0.0001
No ASCVD, CKD, HF	38,549 (57.1%)	206,869 (55.8%)	1,246,613 (58.2%)	1,492,031 (57.8%)	< 0.0001
Baseline comorbidities					
Atrial fibrillation	7015 (10.4%)	34,972 (9.4%)	182,047 (8.5%)	224,034 (8.7%)	< 0.0001
Amputation	795 (1.2%)	4482 (1.2%)	19,580 (0.9%)	24,857 (1.0%)	< 0.0001
Cerebrovascular disease	8255 (12.2%)	48,219 (13.0%)	267,348 (12.5%)	323,822 (12.6%)	< 0.0001
Coronary artery disease	17,099 (25.3%)	94,956 (25.6%)	489,555 (22.9%)	601,610 (23.3%)	< 0.0001
Hypertension	57,640 (85.3%)	321,899 (86.9%)	1,783,787 (83.3%)	2,163,326 (83.9%)	< 0.0001
Hyperglycemic crisis	280 (0.4%)	1887 (0.5%)	11,416 (0.5%)	13,583 (0.5%)	< 0.0001
Hypoglycemic crisis	509 (0.8%)	3395 (0.9%)	16,033 (0.7%)	19,937 (0.8%)	< 0.0001
Neuropathy	16,263 (24.1%)	96,050 (25.9%)	524,136 (24.5%)	636,449 (24.7%)	< 0.0001
Other lower extremity complications	3038 (4.5%)	16,631 (4.5%)	91,909 (4.3%)	111,578 (4.3%)	< 0.0001
Peripheral vascular disease	9128 (13.5%)	55,578 (15.0%)	339,875 (15.9%)	404,581 (15.7%)	< 0.0001
Retinopathy	8320 (12.3%)	48,133 (13.0%)	295,315 (13.8%)	351,768 (13.6%)	< 0.0001
Smoking	8925 (13.2%)	50,509 (13.6%)	223,764 (10.4%)	283,198 (11.0%)	< 0.0001
Lower extremity ulcer	2777 (4.1%)	15,179 (4.1%)	84,336 (3.9%)	102,292 (4.0%)	< 0.0001

**Figure 1:**
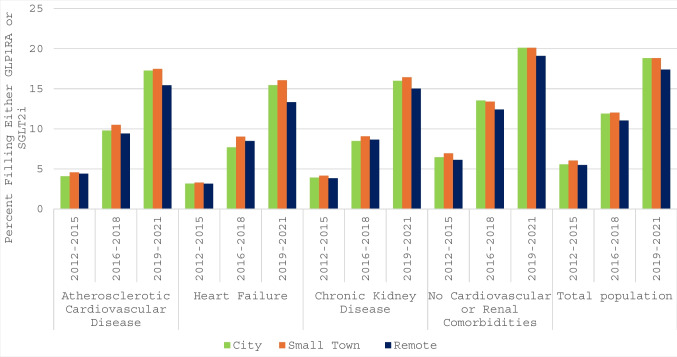
Crude fills of either an SGLT2i or GLP1RA in patients with atherosclerotic cardiovascular disease, heart failure, chronic kidney disease stages 3–4, without these comorbidities, and in the type 2 diabetes population overall between 2012–2015, 2016–2018, and 2019–2021 by level of rurality. GLP1RA, glucagon-like peptide-1 receptor agonists; SGLT2i, sodium-glucose cotransporter-2 inhibitors.

Regardless of comorbidity status or rurality, fills of GLP1RA and SGLT2i increased over time (Fig. 1, Table S10). By comparison to 2012–2015 and adjusted for patient age, sex, rurality, and comorbidities, the odds of GLP1RA or SGLT2i fills were OR 2.49 (95% CI 2.47–2.52) in 2016–2018 and OR 4.35 (95% CI 4.30–4.39) in 2019–2021 (Table S11).

**Figure 2:**
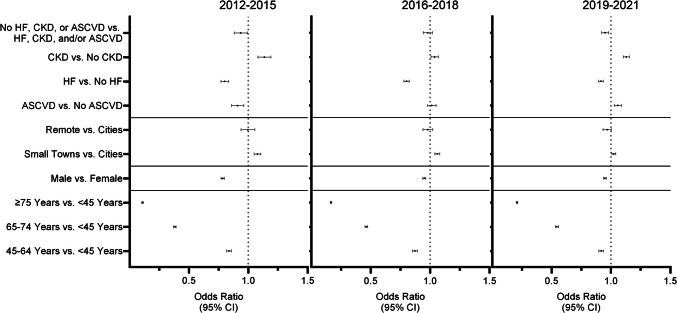
Factors associated with GLP1RA or SGLT2i therapy among adults with type 2 diabetes living in rural and urban areas of the USA. Logistic regression models examined the association between GLP1RA or SGLT2i fills and time period, rurality, age group, sex, and comorbidity status by index year grouping and were adjusted for time period, age, sex, rurality, and cardiovascular and kidney comorbidity as appropriate. ASCVD, atherosclerotic cardiovascular disease; CKD, chronic kidney disease; HF, heart failure

Compared to those aged 18–44, the odds of filling an SGLT2i or GLP1RA in 2012–2015 were OR 0.84 (95% CI 0.82–0.86), OR 0.38 (95% CI 0.37–0.39), and OR 0.11 (95% CI 0.10–0.11) for those aged 45–64, 65–74, and ≥ 75, respectively (Fig. 2). These age-based differences lessened but persisted, to OR 0.92 (95% CI 0.90–0.94), OR 0.54 (95% CI 0.53–0.56), and OR 0.21 (95% CI 0.20–0.21) by 2019–2021. Men were less likely to fill GLP1RA or SGLT2i than women in 2012–2015 (OR 0.78; 95% CI 0.77–0.80), but this gap decreased by 2019–2021 to OR 0.95 (95% CI 0.94–0.96).

There was no statistically significant difference in fills of GLP1RA and SGLT2i between those living in remote areas and cities during the study period; however, those in small towns were likelier to fill these medications than those in cities in both 2012–2015 (OR 1.08; 95% CI 1.05–1.10) and 2019–2021 (OR 1.02; 95% CI 1.01–1.04) time periods.

In 2012–2015, those with ASCVD were less likely to fill a GLP1RA or SGLT2i than those without ASCVD (OR 0.91; 95% CI 0.86–0.96), but this difference dissipated by 2016–2018 and those with ASCVD became more likely to fill GLP1RA or SGLT2i than those without ASCVD by 2019–2021 (OR 1.06; 95% CI 1.03–1.09). Throughout the entire study period, patients with CKD were likelier to fill SGLT2i or GLP1RA than those without CKD, up to OR 1.13 (95% CI 1.10–1.16) in 2019–2021. Conversely, those with HF were less likely to fill GLP1RA or SGLT2i throughout the entire study period, though this gap narrowed over time from OR 0.80 (95% CI 0.77–0.84) in 2012–2015 to OR 0.92 (95% CI 0.90–0.94) in 2019–2021. Those without cardiovascular or kidney comorbidities were slightly less likely to fill SGLT2i or GLP1RA in 2012–2015 (OR 0.94; 95% CI 0.88–0.99) and in 2019–2021 (OR 0.95; 95% CI 0.92–0.98) by comparison to those with these comorbidities. Our results were unchanged in the sensitivity analysis that included insurance type as a covariate. Patients with Medicare Advantage plans filled GLP1RA and SGLT2i less often than those with commercial insurance, though this difference lessened over time (Tables S12-S13).

## DISCUSSION

Between 2012 and 2021, fills of GLP1RA and SGLT2i increased in all patients with type 2 diabetes, and the previously demonstrated benefit/treatment paradox of GLP1RA/SGLT2i use^7^ among patients with ASCVD and kidney comorbidities improved, though gaps remained in HF. Over the study period, patients with ASCVD went from being 9% less likely than those without ASCVD to fill a GLP1RA or SGTL2i in 2012–2015 to being 6% likelier to fill one of these medications in 2019 to 2021; those with CKD were likelier to fill these medications than those without. Patients with HF, men, and older patients were less likely to fill GLP1RA and SGLT2i, but these differences lessened over time. Those in small towns had slightly higher fills than those in cities, and this small difference also narrowed from 8% higher fills between 2012 and 2015 to 2% between 2019 and 2021. Nevertheless, despite improved use of GLP1RA and SGLT2i between 2012 and 2021 overall and in key patient subgroups previously experiencing disparities in access, use of these medications remained low, with fewer than 20% of patients with type 2 diabetes using either a GLP1RA or SGLT2i between 2019 and 2021.

It is reassuring to see the use of GLP1RA and SGLT2i—overall and by patients with ASCVD, CKD, and HF—increasing between 2012 and 2021, as multiple earlier studies revealed concerning gaps in the use of these therapies despite robust evidence of their benefits in reducing major cardiovascular events, progression of kidney disease, and cardiovascular, kidney, and overall mortality.^22,24^ Still, fewer than 1 in 5 patients with type 2 diabetes were using these medications by the end of the study period, signaling a continued need to increase awareness of and access to these evidence-based therapies. Although those with CKD and ASCVD were likelier to use these medications than those without these conditions after controlling for potential confounders, the degree to which they were filled more frequently was marginal, given the use was only 6% higher in those with ASCVD and 13% higher for those with CKD by the end of the study period. Though improved, those with HF still had 8% lower fills of these medications near the end of the study period by comparison to those without HF. These improvements may reflect a response to diabetes guidelines released in 2019, and the trajectory of improvement may have continued since that time, especially since SGLT2i medications became uniformly recommended in HF guidelines in 2022.^25^

There are multiple barriers to optimal use of GLP1RA and SGLT2i drugs. According to a survey of primary care clinicians and specialists at a large academic health care system, endocrinologists and primary care clinicians cited drug costs and the clerical burden of prior authorizations as the biggest barriers to medication initiation. Other specialists such as cardiologists historically did not prescribe diabetes drugs, citing lack of familiarity with or responsibility for a patient’s glycemic status as a major reason for disengagement from optimizing diabetes and cardiovascular risk management.^26^ These barriers will require multi-faceted solutions, including large-scale efforts to reduce the cost of glucose-lowering drugs and clerical burden from prior authorizations, engaging health care team members to share and reduce administrative burden, partnering with pharmacists to support optimal pharmacotherapy selection, titration, and access, and prescriber education on the practical aspects of evidence-based diabetes management.^27^

Although less pronounced with time, the best predictor of GLP1RA and SGLT2i use was young age. Prior studies have shown this trend in prescribing not only among primary care providers but also among nephrologists and endocrinologists.^28^ There may be additional barriers to prescribing GLP1RA and SGLT2i to older adults, including concerns over cost (as Medicare beneficiaries are generally ineligible for copayment reduction coupons) and adverse events, hesitancy to modify treatment regimens in patients with frailty or multimorbidity, as well as uncertainty about the rigor of clinical data available in older adults.^29^ However, it is notable that GLP1RA and SGLT2i fill disparities described here emerged even after controlling for insurance type and prior to the age of 65 in a fully insured cohort, suggesting prescribing decisions are not driven solely by affordability concerns. While concerns about adverse events persist, evidence suggests that in appropriately selected older adults, these medications offer substantial benefits with manageable risks.^30^ Although the incidence of diabetic ketoacidosis is higher in older adults treated with SGLT2i medications, it remains unlikely (29.6/10,000 patient-years) and even less likely among those who eat well and are adequately hydrated.^30,31^ Older adults treated with SGLT2i experience similarly increased risk of genital infections as younger patients (approximately double their baseline risk), and many have low baseline risk.^31^ Older adults are no likelier to suffer from the gastrointestinal side effects of GLP1RA medications; however, these side effects can be more meaningful to older adults, as evidenced by their higher rates of GLP1RA discontinuation.^32^ Nonetheless, GLP1RA medications are generally well-tolerated. Importantly, GLP1RA and SGLT2i medicines are not associated with hypoglycemia (a key consideration in the management of glycemia in older adults) and decrease the risk of cardiovascular events, heart failure, and kidney disease in older adults as much, if not more, than in younger patients.^30^ Therefore, education about safe adjustments of diabetes medication regimens, ongoing research affirming the safety and efficacy of GLP1RA and SGLT2i medications in older adults, and dissemination of this information may help increase their use in this patient demographic.

Patients in rural areas were not less likely than those in cities to fill GLP1RA and SGLT2i medications. In fact, those in small towns were slightly likelier to fill these medicines than those in cities throughout the study period after controlling for potential confounders. Though this difference was small, being 8% higher in small towns than cities between 2012 and 2015 and 2% higher in small towns than cities between 2019 and 2021, the finding was unexpected. Although the health care landscape varies throughout the United States, patients in remote areas tend to lack access to health care, while those in small towns tend to have poor access to subspecialty care, and those in cities tend to have better access to primary care providers and specialists. It is possible that the small town model, which is reliant on individual primary care clinicians who tend to be highly sensitized to diabetes and its consequences,^33^ allowed for increased early uptake of GLP1RA and SGLT2i medications by comparison to cities where there is greater fragmentation of care between subspecialists and primary care providers.^34^ However, this early advantage seems to be lessening in these areas, and rates of GLP1RA and SGLT2i use remained low overall. Thus, interventions to improve uptake of GLP1RA and SGLT2i medications should consider the specific needs of the different health care landscapes that exist in the United States. While cities may benefit from initiatives that enhance coordination among specialists and pharmacists to manage diabetes care, rural health care delivery systems may have different needs. For example, at Rural Health Clinics, non-physician providers deliver care > 50% time by statute, and these clinics may have barriers to delivering GLP1RA and SGLT2i medications that vary from larger health systems.^35^

### Strengths and Limitations

To our knowledge, this is the first study to examine the use of glucose-lowering drugs with cardiovascular and kidney benefits across the rural–urban continuum in a large, diverse national population of adults with type 2 diabetes spanning different health systems, insurance types, and regions. It also provides updated data on the treatment/benefit paradox of glucose-lowering therapies observed previously. However, it has several limitations. These data represent a single national health insurance provider that administers multiple commercial and Medicare Advantage health plans; patients without insurance, those on traditional Medicare fee-for-service insurance, and those using Medicaid are not included. These excluded patients are disproportionately represented in rural areas and experience greater barriers to accessing evidence-based diabetes care and costly drugs.^36^ Moreover, we were not able to assess intersectionality between income, race, ethnicity, and rurality because these data are not all available in our dataset to preserve patient de-identification. Future studies are needed to examine this intersectionality, even if conducted in smaller and less heterogeneous patient populations, particularly given known disparities in access to these newer glucose-lowering agents for those who are Black, underinsured, and have lower income levels.^4,5,37–40^ We also could not examine prescribing of these drugs, as our data represent pharmacy fills and patients may have been prescribed drugs that they subsequently did not or could not fill. Furthermore, we did not have access to laboratory test result data such as hemoglobin A1c, which may have influenced prescribing decisions. Still, these newer diabetes medications are recommended to patients with type 2 diabetes and comorbid ASCVD, HF, and CKD irrespective of their glycemic status.^1^ Finally, we reviewed fill patterns over the course of only 1 year. Further review of fill patterns over longer spans of the disease course would be of interest in future investigations.

## Summary

Patients with type 2 diabetes and ASCVD, HF, or CKD are not commonly treated with highly recommended glucose-lowering medications that have cardiovascular and kidney benefits. Older patients are even less likely to be prescribed these medications. While there was slightly higher use of these medications in small towns by comparison to cities, this difference was small and lessened with time. Understanding context and population-specific barriers to use is essential, and needs in rural areas likely differ from those of urban areas. Future research needs to prioritize quality improvement and implementation science initiatives to address barriers to evidence-based pharmacotherapy across diverse settings and populations.

## Data Sharing Statement

OptumLabs data are subject to limitations on sharing as a condition of access, which is a justifiable reason for limiting sharing under the DMS Policy. The data underlying the results of this study are third-party data owned by OptumLabs and contain sensitive patient information; therefore, the data is only available upon request. Interested researchers engaged in HIPAA-compliant research may contact connected@optum.com for data access requests. The data use requires researchers to pay for rights to use and access the data.

## Supplementary Information

Below is the link to the electronic supplementary material.

Supplementary file1 (DOCX 139 KB)

## References

[1] **ElSayed NA, Aleppo G, Aroda VR, et al.** 9. Pharmacologic Approaches to Glycemic Treatment: Standards of Care in Diabetes—2023. Diabetes Care. 2022;46(Supplement_1):S140-S57. 10.2337/dc23-S009 PMC9810476 36507650

[2] **Morris ZS, Wooding S, Grant J.** The answer is 17 years, what is the question: understanding time lags in translational research. J R Soc Med. 2011 Dec;104(12):510-20. 22179294 10.1258/jrsm.2011.110180 PMC3241518

[3] National Healthcare Quality & Disparities Report Chartbooks. 2022 National Healthcare Quality and Disparities Report. Rockville (MD): Agency for Healthcare Research and Quality (US); 2022.

[4] **McCoy RG, Van Houten HK, Dunlay SM, et al.** Race and sex differences in the initiation of diabetes drugs by privately insured US adults. Endocrine. 2021 Aug;73(2):480-4. 33830439 10.1007/s12020-021-02710-4 PMC8273106

[5] **McCoy RG, Dykhoff HJ, Sangaralingham L, et al.** Adoption of New Glucose-Lowering Medications in the U.S.-The Case of SGLT2 Inhibitors: Nationwide Cohort Study. Diabetes Technol Ther. 2019 Dec;21(12):702-12. 31418588 10.1089/dia.2019.0213 PMC7207017

[6] **McCoy RG, Van Houten HK, Deng Y, et al.** Comparison of Diabetes Medications Used by Adults With Commercial Insurance vs Medicare Advantage, 2016 to 2019. JAMA Netw Open. 2021 Feb 1;4(2):e2035792. 33523188 10.1001/jamanetworkopen.2020.35792 PMC7851726

[7] **McCoy RG, Van Houten HK, Karaca-Mandic P, Ross JS, Montori VM, Shah ND.** Second-Line Therapy for Type 2 Diabetes Management: The Treatment/Benefit Paradox of Cardiovascular and Kidney Comorbidities. Diabetes Care. 2021 Aug 4;44(10):2302-11. 34348996 10.2337/dc20-2977 PMC8929191

[8] **Lamprea-Montealegre JA, Madden E, Tummalapalli SL, et al.** Association of Race and Ethnicity With Prescription of SGLT2 Inhibitors and GLP1 Receptor Agonists Among Patients With Type 2 Diabetes in the Veterans Health Administration System. JAMA. 2022;328(9):861-71. 36066519 10.1001/jama.2022.13885 PMC9449794

[9] **Steiger K, Herrin J, Swarna KS, Davis EM, McCoy RG.** Disparities in Acute and Chronic Complications of Diabetes Along the U.S. Rural-Urban Continuum. Diabetes Care. 2024 May 1;47(5):818-25. 38387066 10.2337/dc23-1552 PMC11043221

[10] **Curtin SC, Spencer MR.** Trends in Death Rates in Urban and Rural Areas: United States, 1999-2019. NCHS Data Brief. 2021 Sep(417):1-8. 34582331

[11] **Grant JS, Steadman LA.** Barriers to Diabetes Self-Management Among Rural Individuals in the Workplace. Workplace Health Saf. 2016 Jun;64(6):243-8. 27016376 10.1177/2165079916628877

[12] **Ross S, Benavides-Vaello S, Schumann L, Haberman M.** Issues that impact type-2 diabetes self-management in rural communities. Journal of the American Association of Nurse Practitioners. 2015 Nov;27(11):653-60. 25776183 10.1002/2327-6924.12225

[13] **Lepard MG, Joseph AL, Agne AA, Cherrington AL.** Diabetes self-management interventions for adults with type 2 diabetes living in rural areas: a systematic literature review. Curr Diab Rep. 2015 Jun;15(6):608. 25948497 10.1007/s11892-015-0608-3 PMC5373659

[14] **Wallace PJ, Shah ND, Dennen T, Bleicher PA, Crown WH.** Optum Labs: building a novel node in the learning health care system. Health Aff (Millwood). 2014 Jul;33(7):1187-94. 25006145 10.1377/hlthaff.2014.0038

[15] HHS. Guidance Regarding Methods for De-identification of Protected Health Information in Accordance with the Health Insurance Portability and Accountability Act (HIPAA) Privacy Rule. Office for Civil Rights. U.S. Department of Health and Human Services (HHS). 2012 [updated November 6, 2015; cited 2019 January 28]; Available from: https://www.hhs.gov/sites/default/files/ocr/privacy/hipaa/understanding/coveredentities/De-identification/hhs_deid_guidance.pdf, https://www.hhs.gov/hipaa/for-professionals/privacy/special-topics/de-identification/index.html#dates.

[16] **von Elm E, Altman DG, Egger M, Pocock SJ, Gøtzsche PC, Vandenbroucke JP.** The Strengthening the Reporting of Observational Studies in Epidemiology (STROBE) statement: guidelines for reporting observational studies. Annals of internal medicine. 2007 Oct 16;147(8):573-7. 17938396 10.7326/0003-4819-147-8-200710160-00010

[17] NCQA. National Committee for Quality Assurance (NCQA) Healthcare Effectiveness Data and Information Set (HEDIS) Comprehensive Diabetes Care. Washington, D.C.: National Committee for Quality Assurance; 2015. p. 74–98.

[18] Documentation: 2010 rural-urban commuting area (RUCA) codes. 2020; Available from: https://www.ers.usda.gov/data-products/rural-urban-commuting-area-codes/documentation/.

[19] Defining Rural Population. Online 2020; Available from: https://www.hhs.gov/guidance/document/defining-rural-population.

[20] **Larson EH AC, Garberson LA, Evans DV.** Geographic Access to Health Care for Rural Medicare Beneficiaries: A National Study2021; 2023(February 12). Available from: https://familymedicine.uw.edu/rhrc/wp-content/uploads/sites/4/2021/09/RHRC_PBSEP2021_LARSON.pdf.

[21] **Zinman B, Wanner C, Lachin JM, et al.** Empagliflozin, Cardiovascular Outcomes, and Mortality in Type 2 Diabetes. New England Journal of Medicine. 2015;373(22):2117-28. 26378978 10.1056/NEJMoa1504720

[22] **Marso SP, Daniels GH, Brown-Frandsen K, et al.** Liraglutide and Cardiovascular Outcomes in Type 2 Diabetes. N Engl J Med. 2016 Jul 28;375(4):311-22. 27295427 10.1056/NEJMoa1603827 PMC4985288

[23] American Diabetes A. 9. Pharmacologic Approaches to Glycemic Treatment: Standards of Medical Care in Diabetes—2019. Diabetes Care. 2018;42(Supplement_1):S90-S102. 10.2337/dc19-S009 30559235

[24] **Zinman B, Wanner C, Lachin JM, et al.** Empagliflozin, Cardiovascular Outcomes, and Mortality in Type 2 Diabetes. N Engl J Med. 2015 Nov 26;373(22):2117-28. 26378978 10.1056/NEJMoa1504720

[25] **Heidenreich PA, Bozkurt B, Aguilar D, et al.** 2022 AHA/ACC/HFSA Guideline for the Management of Heart Failure: A Report of the American College of Cardiology/American Heart Association Joint Committee on Clinical Practice Guidelines. Circulation. 2022 2022/05/03;145(18):e895-e1032. 10.1161/CIR.0000000000001063 35363499

[26] **Gao Y, Peterson E, Pagidipati N.** Barriers to prescribing glucose-lowering therapies with cardiometabolic benefits. Am Heart J. 2020 Jun;224:47-53. 32304879 10.1016/j.ahj.2020.03.017

[27] **Claudel SE, Schmidt IM, Verma A.** A Call for Implementation Science: Achieving Equitable Access to SGLT2 Inhibitors. Kidney360. 2022 May 26;3(5):942-4. 36128478 10.34067/KID.0001512022 PMC9438421

[28] **McCoy RG, Vandergrift JL, Gray B.** Patient and physician factors driving the gaps in use of drugs with cardiovascular and kidney benefits by medicare beneficiaries with type 2 diabetes treated by endocrinologists, nephrologists, and cardiologists: Population-based cohort study. Diabetes Res Clin Pract. 2025 Feb 7;221:112039. 10.1016/j.diabres.2025.112039 39923965

[29] **Mills GB, Ratcovich H, Adams-Hall J, et al.** Is the contemporary care of the older persons with acute coronary syndrome evidence-based? European Heart Journal Open. 2021;2(1). 10.1093/ehjopen/oeab044 PMC9242048 35919658

[30] 13. Older Adults: Standards of Care in Diabetes-2025. Diabetes Care. 2025 Jan 1;48(Supplement_1):S266-s82. 39651977 10.2337/dc25-S013 PMC11635042

[31] **Güdemann LM, Young KG, Thomas NJM, et al.** Safety and effectiveness of SGLT2 inhibitors in a UK population with type 2 diabetes and aged over 70 years: an instrumental variable approach. Diabetologia. 2024 Sep;67(9):1817-27. 38836934 10.1007/s00125-024-06190-9 PMC11410842

[32] **Wang Y, Wang J, Gong Q, et al.** Efficacy and safety of glucagon-like peptide-1 receptor agonists in the elderly versus non-elderly patients with type 2 diabetes mellitus: insights from a systematic review. Endocr J. 2024 Jun 18;71(6):571-82. 38644220 10.1507/endocrj.EJ23-0384

[33] **Bolin JN, Bellamy GR, Ferdinand AO, et al.** Rural Healthy People 2020: New Decade, Same Challenges. J Rural Health. 2015 Summer;31(3):326–33. 10.1111/jrh.12116 25953431

[34] **Stange KC, Miller WL, Etz RS.** The Role of Primary Care in Improving Population Health. Milbank Q. 2023 Apr;101(S1):795-840. 37096603 10.1111/1468-0009.12638 PMC10126984

[35] Rural Health Clinics. Online: Centers for Medicare & Medicaid Services; [updated 03/27/2023 cited 2023 June 25]; Available from: https://www.cms.gov/medicare/provider-enrollment-and-certification/certificationandcomplianc/rhcs.

[36] **Barker AR, Londeree JK, McBride TD, Kemper LM, Mueller K.** The uninsured: an analysis by income and geography. Rural Policy Brief. 2013 Jun 1(2013 6):1–4. 25399459

[37] **Limonte CP, Hall YN, Trikudanathan S, et al.** Prevalence of SGLT2i and GLP1RA use among US adults with type 2 diabetes. J Diabetes Complications. 2022 Jun;36(6):108204. 35537891 10.1016/j.jdiacomp.2022.108204

[38] **Eberly LA, Yang L, Eneanya ND, et al.** Association of Race/Ethnicity, Gender, and Socioeconomic Status With Sodium-Glucose Cotransporter 2 Inhibitor Use Among Patients With Diabetes in the US. JAMA Network Open. 2021;4(4):e216139-e. 33856475 10.1001/jamanetworkopen.2021.6139 PMC8050743

[39] **Mahtta D, Ramsey DJ, Lee MT, et al.** Utilization Rates of SGLT2 Inhibitors and GLP-1 Receptor Agonists and Their Facility-Level Variation Among Patients With Atherosclerotic Cardiovascular Disease and Type 2 Diabetes: Insights From the Department of Veterans Affairs. Diabetes Care. 2022 Feb 1;45(2):372-80. 35015080 10.2337/dc21-1815 PMC8914426

[40] **Kurani SS, Lampman MA, Funni SA, et al.** Association Between Area-Level Socioeconomic Deprivation and Diabetes Care Quality in US Primary Care Practices. JAMA Netw Open. 2021 Dec 1;4(12):e2138438. 34964856 10.1001/jamanetworkopen.2021.38438 PMC8717098

