# Supplementary material for: Fill Patterns of Glucose-Lowering Drugs with Cardiovascular and Kidney Benefits in the Rural and Urban United States, 2012–2021
Source: J Gen Intern Med. 2025 Aug 4;41(1):126–33. doi: 10.1007/s11606-025-09784-0 (PMC12855668; doi:10.1007/s11606-025-09784-0)
Supplement: Supplementary file 1 — Supplementary file1 (DOCX 139 KB) [file 11606_2025_9784_MOESM1_ESM.docx]

**SUPPLEMENTARY MATERIALS**

**Fill Patterns of Glucose-Lowering Drugs with Cardiovascular and Renal Benefits in the Rural and Urban United States, 2012-2021**

Kyle Steiger, MD, Kavya Sindhu Swarna, MPH, Jeph Herrin, PhD, Rozalina G McCoy MD, MS

Table of Contents

[Table S1. International Classification of Disease (ICD)-9 and ICD-10 Codes Used for Exclusion. 2](#_Toc200206783)

[Figure S1. Study Flow Diagram. 3](#_Toc200206784)

[Figure S2. Data Ascertainment Flow Diagram. 4](#_Toc200206785)

[Table S2. International Classification of Disease (ICD)-9 and ICD-10 Codes Used for Baseline Comorbidity Ascertainment. 5](#_Toc200206786)

[Table S3. International Classification of Disease (ICD)-9 and ICD-10 Codes Used for Cardiovascular and Kidney Disease Grouping Determination. 8](#_Toc200206787)

[Table S4. List of Included Medications. 10](#_Toc200206788)

[Table S5. Degree of Comorbidity Grouping Overlap. 11](#_Toc200206789)

[Table S6. Characteristics of group without atherosclerotic cardiovascular disease, heart failure, or CKD stages 3, 4. 12](#_Toc200206790)

[Table S7. Characteristics of group with atherosclerotic cardiovascular disease. 13](#_Toc200206791)

[Table S8. Characteristics of group with heart failure. 14](#_Toc200206792)

[Table S9. Characteristics of group with CKD stages 3 and 4. 15](#_Toc200206793)

[Table S10. Fills of either GLP-1RA or SGLT2i medications, GLP1RA medications, and SGLT2i medications by comorbidity status and level of rurality. 16](#_Toc200206794)

[Table S11. Factors associated with GLP1RA or SGLT2i therapy among adults with type 2 diabetes living in rural and urban areas of the U.S. 18](#_Toc200206795)

[Table S12. Characteristics of patients after excluding patients without insurance information available (n=119,964 excluded). 19](#_Toc200206796)

[Table S13. Factors associated with GLP1RA or SGLT2i therapy among adults with type 2 diabetes living in rural and urban areas of the U.S. with the addition of insurance type as a covariate. 20](#_Toc200206797)

[References Cited 21](#_Toc200206798)

Table S1. International Classification of Disease (ICD)-9 and ICD-10 Codes Used for Exclusion.

These codes were derived from Value Set Authority Center from the National Institutes of Health.(1)

| **Comorbidity** | | **ICD-9 Codes** | **ICD-10 codes** | **CPT codes** | **Revenue Codes** |
| --- | --- | --- | --- | --- | --- |
| **End-stage kidney disease, dialysis, transplantation** | 403.01, 403.11, 403.91, 404.02, 404.03, 404.12, 404.13, 404.92, 404.93, 585.5, 585.6, 792.5, 996.81, V42.0, V45.1, V45.11, V45.12, V56.x, V56.0, V56.1, V56.2, V56.3, V56.31, V56.32, V56.8, 39.95, 54.98, 55.53, 55.6, 55.69 | I12.0, I13.11, I13.2, I95.3, N18.5, N186, R88.0, T81.502x, T81.512x, T81.522x, T81.532x, T81.592x, T85.611x, T85.621x, T85.631x, T85.651x, T85.71x, T86.1, T86.10, T86.11, T86.12, T86.13, T86.19, Y84.1, Z48.22, Z49, Z49.0, Z49.01, Z49.02, Z49.3, Z49.31, Z49.32, Z91.15, Z94.0, Z99.2, 0TT00ZZ, 0TT04ZZ, 0TT10ZZ, 0TT14ZZ, 0TT30ZZ, 0TT34ZZ, 0TT37ZZ, 0TT38ZZ, 0TT40ZZ, 0TT44ZZ, 0TY00Z0, 0TY00Z1, 0TY00Z2, 0TY10Z0, 0TY10Z1, 0TY10Z2, 3E1M39Z, 5A1D00Z, 5A1D60Z  Dialysis ICD 10 PCS – 5A1D70Z, 5A1D80Z, 5A1D90Z | 50340, 50360, 50365, 50370, 90935, 90937, 90940, 90945, 90947, 90957, 90958, 90959, 90960, 90961, 90962, 90965, 90966, 90969, 90970, 90999, G0257, S9335, 99512  Dialysis CPT – 90921, 90925, 90991, 90992, 90994 | 0800, 0801, 0802, 0803, 0804, 0805, 0806, 0807, 0808, 0809, 0820, 0821, 0822, 0823, 0824, 0825, 0826, 0827, 0828, 0829, 0830, 0831, 0832, 0833, 0834, 0835, 0836, 0837, 0838, 0839, 0840, 0841, 0842, 0843, 0844, 0845, 0846, 0847, 0848, 0849, 0850, 0851, 0852, 0853, 0854, 0855, 0856, 0857, 0858, 0859, 0880, 0881, 0882, 0883, 0884, 0885, 0886, 0887, 0888, 0889 |  |
| **Type 1 diabetes** |  | E10.x, O240.x, ICD9: 250.x1, 250.x3 |  |  |  |

# Figure S1. Study Flow Diagram.

Adults in OptumLabs® with adequate follow up, type 2 diabetes, geographic information, and without end-stage kidney disease.

(n=2,579,577)

Patients with type 1 diabetes

(n=268,429)

Patients without geographic data

(n=7,337)

Patients less than 18 years of age or without age information

(n=27,275)

Patients without 12 months of follow up

(n=1,231,273)

Adults in OptumLabs® with adequate follow up, diabetes, and geographic data

(n=2,901,563)

Adults in OptumLabs® with adequate follow up, type 2 diabetes, and geographic data

(n=2,633,134)

Adults in Optumlabs® with adequate follow up and diabetes

(n=2,908,900)

Patients included in OptumLabs® with diabetes and adequate follow up

(n=2,936,175)

Patients included in OptumLabs® Data Warehouse with diabetes between January 1, 2011 and December 31, 2020

(n=4,167,448)

Patients with end-stage kidney disease

(n=53,557)

# Figure S2. Data Ascertainment Flow Diagram.


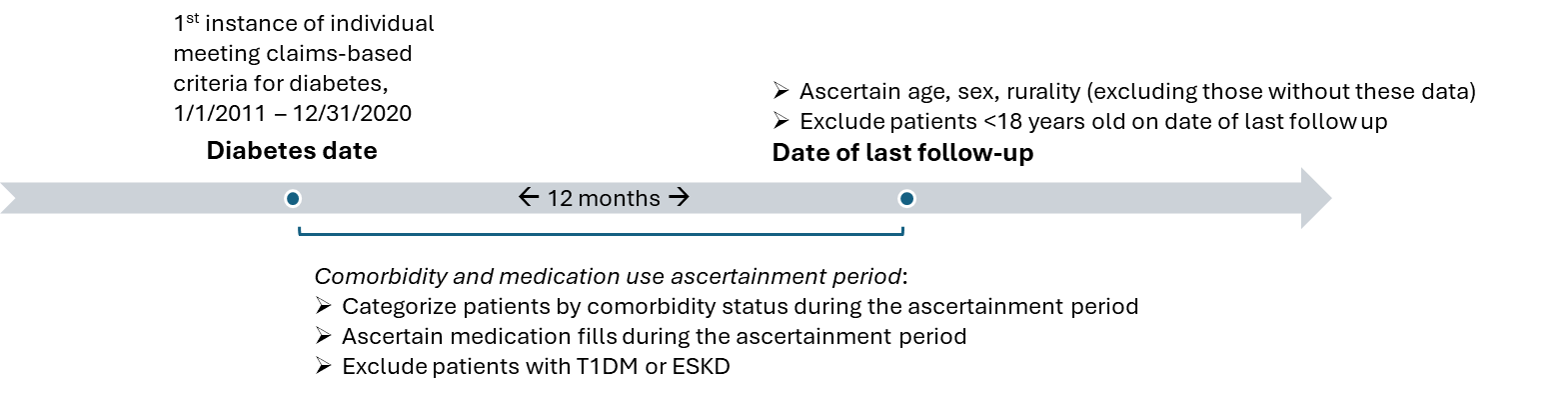


Table S2. International Classification of Disease (ICD)-9 and ICD-10 Codes Used for Baseline Comorbidity Ascertainment. These codes were generally derived from Value Set Authority Center from the National Institutes of Health(1) unless otherwise cited below wherein they were derived from code sets used in prior publications. For example, in the case of hypoglycemia, we utilized the validated Ginde algorithm.(2)

| **Comorbidity** | **ICD-9 Codes** | **ICD-10 codes** | **CPT codes** |
| --- | --- | --- | --- |
| **Atrial fibrillation and flutter** | 427.31, 427.32 | I48.0, I48.1x, I48.2x, I48.91,  I48.3, I48.4, I48.92 |  |
| **Cerebrovascular disease** | 430, 431, 432.x, 433.xx, 434.xx 435.x, 436, 437.x, 438.xx, V12.54 | G45.0, G45.1, G45.2, G45.8, G45.9, G46.x, I60.xx, I61.x (except I61.0), I62.xx, I63.xxx, I65.xx, I66.xx, I67.8x (except I67.83, I67.84), I67.9, I69.xxx, Z86.73 |  |
| **Coronary artery disease** | 429.2, 410.x, 411.x, 412.x, 413.x, 414.x | I20.x, I21.x, I22.x, I23.x, I24x, I25.x |  |
| **Chronic kidney disease stages 3-4** | 585.3 (stage 3), 585.4 (stage 4) | N18.3 (stage 3), N18.4 (stage 4) |  |
| **Heart failure** | 398.91, 402.01, 402.11, 402.91, 404.01, 404.03, 404.11, 404.13, 404.91, 404.93, 428.xx | I09.81, I11.0, I13.0, I13.2, I50.xx |  |
| **Hypoglycemia** | 251.0, 251.1, 251.2, 962.3, 250.8x (for 250.8x: if no concurrent 259.8, 272.7, 681.xx, 682.xx, 686.9x, 707.1x-707.2x, 707.8, 707.9, 709.3, 730.0x-730.2x, 731.8 on the same date)  *DX1 in ED or admission hospital claim* | E10.641, E10.649, E11.641, E11.649, E13.641, E13.649, E16.0, E16.1, E16.2, T38.3X1A, T38.3X1D, T38.3X1S, T38.3X2A, T38.3X2D, T38.3X2S, T38.3X3A, T38.3X3D, T38.3X3S, T38.3X4A, T383X4D, T38.3X4S, T38.3X5A, T383X5D, T38.3X5S  *DX1 in ED or admission hospital claim* |  |
| **Hyperglycemia** | 250.10, 250.11, 250.12, 250.13, 250.20, 250.21, 250.22, 250.23  *DX1 in ED or admission hospital claim* | E10.10, E10.11, E11.10, E11.11, E13.10, E13.11, E11.00, E11.01, E13.00, E13.01  *DX1 in ED or admission hospital claim* |  |
| **Hypertension** | 401.x, 402.xx, 403.xx, 404.xx, 405.xx | I10, I11.x, I12.x, I13.xx, I15.x, I16.x |  |
| **Lower extremity amputation**(3) | Amputation status: V49.7  Amputation: ICD9 procedure codes 84.10-84.19 | Amputation status: Z89.4x-Z89.6x | Amputation procedure: 27590, 27880, 27889, 27888, 27882, 27884, 27886, 28120, 28122, 28124, 28800, 28805, 28810, 28820, or 28825 |
| **Other lower extremity complications:** Foot/leg osteomyelitis, ulcer,(3) Charcot arthropathy(4, 5) | Osteomyelitis: 730.0x, 730.1x, 730.2, 730.8x, 730.9x    Ulcer: 707.0x, 707.1x, 707.2x, 707.9    Charcot arthropathy: 713.5 | Osteomyelitis: M01.xx, M86.xx    Ulcer: L89.5xx, L89.6xxx, L97.2xxx, L97.3xxx, L97.4xxx, L97.5xxx, L97.8xxx, L97.9xxx    Charcot arthropathy: M14.6x |  |
| **Neuropathy** | 357.2, 337.1, 356.9, 358.1, 458.0, 536.3, 564.5, 596.54, 713.5, 951.0, 951.1, 951.3, 250.6x, 249.6x, 337.0x, 354.x, 355.x | G90.09, G90.8, G90.9, G99.0, G60.9, G73.3, G90.01, I95.1, K31.84, K59.1, N31.9, E08.4x, E09.4x, E10.4x, E11.4x, E13.4x, G56.x, G57.x, H49.x, M14.6x, S04.x |  |
| **Peripheral vascular disease** | 442.3, 440.21, 443.81, 443.9, 892.1, 040.0, 444.22, 785.4, 250.7x, 249.7, 707.1x | E08.51, E09.51, E10.51, E11.51, E13.51, E08.59, E09.59, E10.59, E11.59, E13.59, E08.621, E09.621, E10.621, E11.621, E13.621, I72.4, I73.89, I73.9, A48.0, I74.3, I96, E08.52, E09.52, E10.52, E11.52, E13.52, I70.21x, S91.3x, L97.x |  |
| **Revascularization procedure** | CABG:  Procedure: 36.1x  DX: V45.81, 414.02, 414.03, 414.04, 414.05  PCI:  DX: V45.82  Procedure: 36.0x, 00.6x (*includes coronary and other*), 00.4x (*includes coronary and other*)  LIMB: 39.25, 39.29, 38.08, 38.16, 38.18, 38.38, 38.48, 38.68, 38.88, 39.50, 39.90, 00.55, 84.3, 84.1x | CABG:  Procedure: 02100x, 02110x, 02120x, 02130x  DX: Z95.1, T82.21x, I25.7x (except I25.75x), I25.810, I25.812  PCI:  DX: Z98.61, Z95.5, V45.82  Procedure: 0270x, 0271x, 0272x, 0273x, 02C0x, 02C1x, 02C2x, 02C3x  LIMB: 041x, 047x, 04Bx, 04Cx, 04Lx, 04Px, 04Rx (4^th^ letter C-Y, except G, I, O, X) | CABG: 33510, 33511, 33512, 33513, 33514, 33516, 33517, 33518, 33519, 33521, 33522, 33523, 33533, 33534, 33535, 33536, 4110F  PCI: 92920, 92921, 92924, 92925, 92928, 92929, 92933, 92934, 92937, 92938, 92941, 92943, 92944, 92980, 92981, 92982, 92984, 92995, 92996, 92975, 92977 |
| **Retinopathy** | 362.01, 362.03, 362.04, 362.05, 362.06, 362.07, 362.53, 362.81, 362.82, 362.83, 362.02, 379.23, 250.5x, 249.5x, 362.1x, 361.x, 369.x | H35.9, E08.3x, E09.3x, E10.3x, E11.3x, E13.3x, H35.0x, H35.35x, H35.6x, H35.8x, H33.x, H54.x, H43.1x |  |
| **Smoking** | 305.1, 649.0x, 989.84 | F17.xx (except F17.2x1), Z72.0, O99.33x, T65.2x, Z53.01, Z71.6, | 4000F, 4001F, 4004F, 99406, 99407, C9801, C9802, G0375, G0376, G0436, G0437, G8402, G8453, G8455, G9276, G9458, G9792 |

# Table S3. International Classification of Disease (ICD)-9 and ICD-10 Codes Used for Cardiovascular and Kidney Disease Grouping Determination.

These codes were derived from Value Set Authority Center from the National Institutes of Health.(1)

| **Comorbidity** | **ICD-9 Codes** | **ICD-10 codes** | **CPT codes** |
| --- | --- | --- | --- |
| **Atherosclerotic cardiovascular disease** | 429.2, 410.x, 411.x, 412.x, 413.x, 414.x  430, 431, 432.x, 433.xx, 434.xx 435.x, 436, 437.x, 438.xx, V12.54  442.3, 440.21, 443.81, 443.9, 892.1, 040.0, 444.22, 785.4, 250.7x, 249.7, 707.1x  CABG:  Procedure: 36.1x  DX: V45.81, 414.02, 414.03, 414.04, 414.05    PCI:  DX: V45.82  Procedure: 36.0x, 00.6x (*includes coronary and other*), 00.4x (*includes coronary and other*)    LIMB: 39.25, 39.29, 38.08, 38.16, 38.18, 38.38, 38.48, 38.68, 38.88, 39.50, 39.90, 00.55, 84.3, 84.1x | I20.x, I21.x, I22.x, I23.x, I24x, I25.x  G45.0, G45.1, G45.2, G45.8, G45.9, G46.x, I60.xx, I61.x (except I61.0), I62.xx, I63.xxx, I65.xx, I66.xx, I67.8x (except I67.83, I67.84), I67.9, I69.xxx, Z86.73  E08.51, E09.51, E10.51, E11.51, E13.51, E08.59, E09.59, E10.59, E11.59, E13.59, E08.621, E09.621, E10.621, E11.621, E13.621, I72.4, I73.89, I73.9, A48.0, I74.3, I96, E08.52, E09.52, E10.52, E11.52, E13.52, I70.21x, S91.3x, L97.x  CABG:  Procedure: 02100x, 02110x, 02120x, 02130x  DX: Z95.1, T82.21x, I25.7x (except I25.75x), I25.810, I25.812  PCI:  DX: Z98.61, Z95.5, V45.82  Procedure: 0270x, 0271x, 0272x, 0273x, 02C0x, 02C1x, 02C2x, 02C3x  LIMB: 041x, 047x, 04Bx, 04Cx, 04Lx, 04Px, 04Rx (4^th^ letter C-Y, except G, I, O, X) | CABG: 33510, 33511, 33512, 33513, 33514, 33516, 33517, 33518, 33519, 33521, 33522, 33523, 33533, 33534, 33535, 33536, 4110F  PCI: 92920, 92921, 92924, 92925, 92928, 92929, 92933, 92934, 92937, 92938, 92941, 92943, 92944, 92980, 92981, 92982, 92984, 92995, 92996, 92975, 92977 |
| **Heart failure** | 398.91, 402.01, 402.11, 402.91, 404.01, 404.03, 404.11, 404.13, 404.91, 404.93, 428.xx | I09.81, I11.0, I13.0, I13.2, I50.xx |  |
| **Chronic kidney disease stages 3,4** | 585.3 (stage 3), 585.4 (stage 4) | N18.3 (stage 3), N18.4 (stage 4) |  |

Table S4. List of Included Medications.

| Medication Class | Included Agents |
| --- | --- |
| Glucagon-like peptide-1 (GLP-1) receptor agonists | Exenatide  Liraglutide  Albiglutide  Dulaglutide  Semaglutide  Lixisenatide |
| Sodium-glucose transport protein 2 (SGLT-2) inhibitors | Canagliflozin  Empagliflozin  Dapagliflozin  Ertugliflozin |

# Table S5. Degree of Comorbidity Grouping Overlap.

|  | N (%) |
| --- | --- |
| Patients with only vascular disease | 628,564 (24.37%) |
| Patients with only heart failure | 44,716 (1.73%) |
| Patients with only chronic kidney disease stages 3 and 4 | 85,439 (3.31%) |
| Patients with vascular disease and heart failure | 157,664 (6.11%) |
| Patients with vascular disease and chronic kidney disease stages 3 and 4 | 91,657 (3.55%) |
| Patients with heart failure and chronic kidney disease stages 3 and 4 | 11,876 (0.46%) |
| Patients with vascular disease, heart failure, and chronic kidney disease stages 3 and 4 | 67,630 (2.62%) |

# Table S6. Characteristics of group without atherosclerotic cardiovascular disease, heart failure, or CKD stages 3, 4.

|  | **Remote**  **(N=38,549)** | **Small Town**  **(N=206,869)** | **City**  **(N=1,246,613)** | **P-Value** |
| --- | --- | --- | --- | --- |
| **Age in years, mean (SD)** | 62.2 (11.9) | 61.3 (12.2) | 59.9 (12.5) | <0.0001 |
| **Age groups, years** |  |  |  | <0.0001 |
| 18-44 | 3136 (8.1%) | 20468 (9.9%) | 152669 (12.2%) |  |
| 45-64 | 16997 (44.1%) | 93226 (45.1%) | 583069 (46.8%) |  |
| 65-74 | 12853 (33.3%) | 65974 (31.9%) | 368068 (29.5%) |  |
| >75 | 5563 (14.4%) | 27201 (13.1%) | 142807 (11.5%) |  |
| **Sex** |  |  |  | <0.0001 |
| Female | 19445 (50.4%) | 109051 (52.7%) | 633396 (50.8%) |  |
| Male | 19104 (49.6%) | 97818 (47.3%) | 613217 (49.2%) |  |
| **Comorbidities** |  |  |  |  |
| Atrial fibrillation | 1405 (3.6%) | 6433 (3.1%) | 32625 (2.6%) | <0.0001 |
| Amputation | 65 (0.2%) | 436 (0.2%) | 1699 (0.1%) | <0.0001 |
| Hypertension | 30365 (78.8%) | 166355 (80.4%) | 945205 (75.8%) | <0.0001 |
| Hyperglycemic crisis | 140 (0.4%) | 863 (0.4%) | 5817 (0.5%) | 0.0002 |
| Hypoglycemic crisis | 116 (0.3%) | 854 (0.4%) | 4235 (0.3%) | <0.0001 |
| Neuropathy | 6307 (16.4%) | 36197 (17.5%) | 203243 (16.3%) | <0.0001 |
| Other lower extremity  complications | 147 (0.4%) | 787 (0.4%) | 4978 (0.4%) | 0.4012 |
| Retinopathy | 3649 (9.5%) | 20622 (10.0%) | 129460 (10.4%) | <0.0001 |
| Smoking | 4016 (10.4%) | 22301 (10.8%) | 105213 (8.4%) | <0.0001 |
| Lower extremity ulcer | 56 (0.1%) | 320 (0.2%) | 2407 (0.2%) | 0.0001 |
| **Diabetes medications** |  |  |  |  |
| Any insulin | 7014 (18.2%) | 37695 (18.2%) | 209046 (16.8%) | <0.0001 |
| Intermediate/long-acting insulin | 6371 (16.5%) | 33880 (16.4%) | 187093 (15.0%) | <0.0001 |
| Short/rapid-acting insulin | 3076 (8.0%) | 16434 (7.9%) | 97502 (7.8%) | 0.094 |
| Metformin | 25101 (65.1%) | 135302 (65.4%) | 813378 (65.2%) | 0.31 |
| Sulfonylureas | 11285 (29.3%) | 60455 (29.2%) | 335982 (27.0%) | <0.0001 |
| GLP-1 receptor agonists | 3048 (7.9%) | 17644 (8.5%) | 98440 (7.9%) | <0.0001 |
| SGLT2 inhibitors | 2254 (5.8%) | 12971 (6.3%) | 73442 (5.9%) | <0.0001 |
| DPP4-inhibitors | 4433 (11.5%) | 26258 (12.7%) | 165105 (13.2%) | <0.0001 |
| Glinides | 163 (0.4%) | 904 (0.4%) | 7784 (0.6%) | <0.0001 |
| Thiazolidinediones | 2801 (7.3%) | 14852 (7.2%) | 90357 (7.2%) | 0.524 |
| Other diabetes medications | 94 (0.2%) | 544 (0.3%) | 3752 (0.3%) | 0.0023 |
| **Cardiovascular medications** |  |  |  |  |
| Anticoagulants | 1516 (3.9%) | 7343 (3.5%) | 37049 (3.0%) | <0.0001 |
| Antiplatelet medications | 385 (1.0%) | 2326 (1.1%) | 10187 (0.8%) | <0.0001 |
| Antihypertensives | 28056 (72.8%) | 152450 (73.7%) | 859600 (69.0%) | <0.0001 |
| Lipid lowering medications | 22403 (58.1%) | 120996 (58.5%) | 738456 (59.2%) | <0.0001 |

# **Table S7.** Characteristics of group with atherosclerotic cardiovascular disease.

|  | **Remote**  **(N=24,903)** | **Small Towns**  **(N=141,626)** | **City**  **(N=778,986)** | **P-Value** |
| --- | --- | --- | --- | --- |
| **Age in years, mean (SD)** | 69.3 (9.8) | 68.8 (10.1) | 69.0 (10.4) | <0.0001 |
| **Age groups, years** |  |  |  | <0.0001 |
| 18-44 | 340 (1.4%) | 2452 (1.7%) | 16129 (2.1%) |  |
| 45-64 | 6646 (26.7%) | 40211 (28.4%) | 211780 (27.2%) |  |
| 65-74 | 9764 (39.2%) | 54787 (38.7%) | 294612 (37.8%) |  |
| >75 | 8153 (32.7%) | 44176 (31.2%) | 256465 (32.9%) |  |
| **Sex** |  |  |  | <0.0001 |
| Female | 10596 (42.5%) | 66231 (46.8%) | 360010 (46.2%) |  |
| Male | 14307 (57.5%) | 75395 (53.2%) | 418976 (53.8%) |  |
| **Comorbidities** |  |  |  |  |
| Atrial fibrillation | 4926 (19.8%) | 25271 (17.8%) | 133414 (17.1%) | <0.0001 |
| Amputation | 716 (2.9%) | 3939 (2.8%) | 17551 (2.3%) | <0.0001 |
| Chronic kidney disease stages 3, 4 | 4105 (16.5%) | 24384 (17.2%) | 130798 (16.8%) | <0.0001 |
| Hypertension | 23442 (94.1%) | 134427 (94.9%) | 729276 (93.6%) | <0.0001 |
| Hyperglycemic crisis | 123 (0.5%) | 898 (0.6%) | 4862 (0.6%) | 0.0302 |
| Hypoglycemic crisis | 346 (1.4%) | 2279 (1.6%) | 10625 (1.4%) | <0.0001 |
| Neuropathy | 8862 (35.6%) | 53492 (37.8%) | 288189 (37.0%) | <0.0001 |
| Heart failure | 6463 (26.0%) | 37037 (26.2%) | 181794 (23.3%) | <0.0001 |
| Other lower extremity complications | 2847 (11.4%) | 15604 (11.0%) | 85518 (11.0%) | 0.0755 |
| Retinopathy | 4034 (16.2%) | 24045 (17.0%) | 145975 (18.7%) | <0.0001 |
| Smoking | 4458 (17.9%) | 25770 (18.2%) | 108172 (13.9%) | <0.0001 |
| Lower extremity ulcer | 2696 (10.8%) | 14733 (10.4%) | 81097 (10.4%) | 0.1045 |
| **Diabetes medications** |  |  |  |  |
| Any insulin | 7106 (28.5%) | 42204 (29.8%) | 215748 (27.7%) | <0.0001 |
| Intermediate/long-acting insulin | 6404 (25.7%) | 37519 (26.5%) | 190274 (24.4%) | <0.0001 |
| Short/rapid-acting insulin | 3818 (15.3%) | 22493 (15.9%) | 122885 (15.8%) | 0.0866 |
| Metformin | 13316 (53.5%) | 75984 (53.7%) | 414236 (53.2%) | 0.0035 |
| Sulfonylureas | 7199 (28.9%) | 41393 (29.2%) | 214823 (27.6%) | <0.0001 |
| GLP-1 receptor agonists | 1672 (6.7%) | 10363 (7.3%) | 50112 (6.4%) | <0.0001 |
| SGLT2 inhibitors | 1172 (4.7%) | 7649 (5.4%) | 36051 (4.6%) | <0.0001 |
| DPP4-inhibitors | 2760 (11.1%) | 17567 (12.4%) | 100510 (12.9%) | <0.0001 |
| Glinides | 119 (0.5%) | 868 (0.6%) | 7790 (1.0%) | <0.0001 |
| Thiazolidinediones | 1368 (5.5%) | 7862 (5.6%) | 45117 (5.8%) | 0.0004 |
| Other diabetes medications | 85 (0.3%) | 459 (0.3%) | 2972 (0.4%) | 0.0035 |
| **Cardiovascular medications** |  |  |  |  |
| Anticoagulants | 3953 (15.9%) | 20998 (14.8%) | 110152 (14.1%) | <0.0001 |
| Antiplatelet medications | 6247 (25.1%) | 36667 (25.9%) | 175832 (22.6%) | <0.0001 |
| Antihypertensives | 21395 (85.9%) | 125016 (88.3%) | 678105 (87.0%) | <0.0001 |
| Lipid lowering medications | 18494 (74.3%) | 107277 (75.7%) | 592240 (76.0%) | <0.0001 |

# Table S8. Characteristics of group with heart failure.

|  | **Remote**  **(N=8,275)** | **Small Town**  **(N=46,695)** | **City**  **(N=226,916)** | **P-Value** |
| --- | --- | --- | --- | --- |
| **Age, mean (SD)** | 70.9 (9.8) | 70.2 (10.0) | 70.7 (10.3) | <0.0001 |
| **Age groups, years** |  |  |  | <0.0001 |
| 18-44 | 99 (1.2%) | 629 (1.3%) | 3582 (1.6%) |  |
| 45-64 | 1839 (22.2%) | 11597 (24.8%) | 52300 (23.0%) |  |
| 65-74 | 3061 (37.0%) | 17095 (36.6%) | 78373 (34.5%) |  |
| >75 | 3276 (39.6%) | 17374 (37.2%) | 92661 (40.8%) |  |
| **Sex** |  |  |  | <0.0001 |
| Female | 3838 (46.4%) | 23854 (51.1%) | 114810 (50.6%) |  |
| Male | 4437 (53.6%) | 22841 (48.9%) | 112106 (49.4%) |  |
| **Comorbidities** |  |  |  |  |
| Atrial fibrillation | 3117 (37.7%) | 16011 (34.3%) | 79990 (35.3%) | <0.0001 |
| Amputation | 251 (3.0%) | 1472 (3.2%) | 5872 (2.6%) | <0.0001 |
| Chronic kidney disease stages 3, 4 | 2257 (27.3%) | 13028 (27.9%) | 64221 (28.3%) | 0.0347 |
| Cerebrovascular disease | 2264 (27.4%) | 13747 (29.4%) | 69182 (30.5%) | <0.0001 |
| Coronary artery disease | 5397 (65.2%) | 30603 (65.5%) | 144900 (63.9%) | <0.0001 |
| Hypertension | 7991 (96.6%) | 45496 (97.4%) | 220070 (97.0%) | <0.0001 |
| Hyperglycemic crisis | 70 (0.8%) | 369 (0.8%) | 1869 (0.8%) | 0.7371 |
| Hypoglycemic crisis | 168 (2.0%) | 1078 (2.3%) | 4766 (2.1%) | 0.0144 |
| Neuropathy | 3168 (38.3%) | 19327 (41.4%) | 92006 (40.5%) | <0.0001 |
| Other lower extremity complications | 958 (11.6%) | 5428 (11.6%) | 28578 (12.6%) | <0.0001 |
| Peripheral vascular disease | 2543 (30.7%) | 15689 (33.6%) | 85999 (37.9%) | <0.0001 |
| Retinopathy | 1457 (17.6%) | 8446 (18.1%) | 45756 (20.2%) | <0.0001 |
| Revascularization | 2329 (28.1%) | 12560 (26.9%) | 54889 (24.2%) | <0.0001 |
| Smoking | 1527 (18.5%) | 8911 (19.1%) | 33599 (14.8%) | <0.0001 |
| Lower extremity ulcer | 896 (10.8%) | 5072 (10.9%) | 26872 (11.8%) | <0.0001 |
| **Diabetes medications** |  |  |  |  |
| Any insulin | 2764 (33.4%) | 16500 (35.3%) | 77502 (34.2%) | <0.0001 |
| Intermediate/long-acting insulin | 2470 (29.8%) | 14612 (31.3%) | 68093 (30.0%) | <0.0001 |
| Short/rapid-acting insulin | 1618 (19.6%) | 9399 (20.1%) | 47955 (21.1%) | <0.0001 |
| Metformin | 3688 (44.6%) | 21040 (45.1%) | 98537 (43.4%) | <0.0001 |
| Sulfonylureas | 2302 (27.8%) | 13115 (28.1%) | 59975 (26.4%) | <0.0001 |
| GLP-1 receptor agonists | 493 (6.0%) | 3169 (6.8%) | 13200 (5.8%) | <0.0001 |
| SGLT2 inhibitors | 324 (3.9%) | 2133 (4.6%) | 8411 (3.7%) | <0.0001 |
| DPP4-inhibitors | 859 (10.4%) | 5545 (11.9%) | 27355 (12.1%) | <0.0001 |
| Glinides | 37 (0.4%) | 311 (0.7%) | 2451 (1.1%) | <0.0001 |
| Thiazolidinediones | 381 (4.6%) | 1896 (4.1%) | 8959 (3.9%) | 0.0075 |
| Other diabetes medications | 28 (0.3%) | 166 (0.4%) | 802 (0.4%) | 0.971 |
| **Cardiovascular medications** |  |  |  |  |
| Anticoagulants | 2289 (27.7%) | 12387 (26.5%) | 62211 (27.4%) | 0.0003 |
| Antiplatelet medications | 1922 (23.2%) | 11580 (24.8%) | 51485 (22.7%) | <0.0001 |
| Antihypertensives | 7402 (89.5%) | 43525 (93.2%) | 212274 (93.5%) | <0.0001 |
| Lipid lowering medications | 5871 (70.9%) | 34425 (73.7%) | 169063 (74.5%) | <0.0001 |

# Table S9. Characteristics of group with CKD stages 3 and 4.

|  | **Remote**  **(N=6,769)** | **Small Town**  **(N=38,869)** | **City**  **(N=210,964)** | **P-Value** |
| --- | --- | --- | --- | --- |
| **Age, mean (SD)** | 72.6 (9.0) | 71.9 (9.2) | 72.1 (9.4) | <0.0001 |
| **Age groups, years** |  |  |  | <0.0001 |
| 18-44 | 46 (0.7%) | 286 (0.7%) | 1908 (0.9%) |  |
| 45-64 | 1061 (15.7%) | 6968 (17.9%) | 36527 (17.3%) |  |
| 65-74 | 2602 (38.4%) | 15327 (39.4%) | 79936 (37.9%) |  |
| >75 | 3060 (45.2%) | 16288 (41.9%) | 92593 (43.9%) |  |
| **Sex** |  |  |  | <0.0001 |
| Female | 3345 (49.4%) | 20720 (53.3%) | 107441 (50.9%) |  |
| Male | 3424 (50.6%) | 18149 (46.7%) | 103523 (49.1%) |  |
| **Comorbidities** |  |  |  |  |
| Atrial fibrillation | 1494 (22.1%) | 7844 (20.2%) | 41241 (19.5%) | <0.0001 |
| Amputation | 198 (2.9%) | 1141 (2.9%) | 5152 (2.4%) | <0.0001 |
| Cerebrovascular disease | 1543 (22.8%) | 9479 (24.4%) | 50377 (23.9%) | 0.0085 |
| Coronary artery disease | 3033 (44.8%) | 17397 (44.8%) | 88148 (41.8%) | <0.0001 |
| Hypertension | 6554 (96.8%) | 37921 (97.6%) | 204268 (96.8%) | <0.0001 |
| Hyperglycemic crisis | 48 (0.7%) | 334 (0.9%) | 1636 (0.8%) | 0.1749 |
| Hypoglycemic crisis | 144 (2.1%) | 974 (2.5%) | 4389 (2.1%) | <0.0001 |
| Neuropathy | 2576 (38.1%) | 15548 (40.0%) | 84379 (40.0%) | 0.0056 |
| Heart failure | 2257 (33.3%) | 13028 (33.5%) | 64221 (30.4%) | <0.0001 |
| Other lower extremity complications | 638 (9.4%) | 3774 (9.7%) | 20297 (9.6%) | 0.7307 |
| Peripheral vascular disease | 1837 (27.1%) | 11476 (29.5%) | 67802 (32.1%) | <0.0001 |
| Retinopathy | 1392 (20.6%) | 8193 (21.1%) | 48175 (22.8%) | <0.0001 |
| Revascularization | 1257 (18.6%) | 6472 (16.7%) | 30848 (14.6%) | <0.0001 |
| Smoking | 805 (11.9%) | 4831 (12.4%) | 21081 (10.0%) | <0.0001 |
| Lower extremity ulcer | 587 (8.7%) | 3480 (9.0%) | 18826 (8.9%) | 0.7526 |
| **Diabetes medications** |  |  |  |  |
| Any insulin | 2343 (34.6%) | 14697 (37.8%) | 74248 (35.2%) | <0.0001 |
| Intermediate/long-acting insulin | 2085 (30.8%) | 13026 (33.5%) | 65958 (31.3%) | <0.0001 |
| Short/rapid-acting insulin | 1342 (19.8%) | 8199 (21.1%) | 43455 (20.6%) | 0.02 |
| Metformin | 2306 (34.1%) | 13222 (34.0%) | 74283 (35.2%) | <0.0001 |
| Sulfonylureas | 2044 (30.2%) | 12203 (31.4%) | 64363 (30.5%) | 0.0017 |
| GLP-1 receptor agonists | 531 (7.8%) | 3258 (8.4%) | 15829 (7.5%) | <0.0001 |
| SGLT2 inhibitors | 247 (3.6%) | 1553 (4.0%) | 7464 (3.5%) | <0.0001 |
| DPP4-inhibitors | 885 (13.1%) | 5999 (15.4%) | 32548 (15.4%) | <0.0001 |
| Glinides | 52 (0.8%) | 383 (1.0%) | 3051 (1.4%) | <0.0001 |
| Thiazolidinediones | 410 (6.1%) | 2557 (6.6%) | 14066 (6.7%) | 0.1222 |
| Other diabetes medications | 33 (0.5%) | 157 (0.4%) | 1002 (0.5%) | 0.1603 |
| **Cardiovascular medications** |  |  |  |  |
| Anticoagulants | 1149 (17.0%) | 6372 (16.4%) | 33672 (16.0%) | 0.0115 |
| Antiplatelet medications | 1176 (17.4%) | 7215 (18.6%) | 35256 (16.7%) | <0.0001 |
| Antihypertensives | 5941 (87.8%) | 35753 (92.0%) | 194915 (92.4%) | <0.0001 |
| Lipid lowering medications | 4891 (72.3%) | 29265 (75.3%) | 162032 (76.8%) | <0.0001 |

# Table S10. Fills of either GLP-1RA or SGLT2i medications, GLP1RA medications, and SGLT2i medications by comorbidity status and level of rurality.

|  |  | **City** | **Small Town** | | **Remote** |
| --- | --- | --- | --- | --- | --- |
|  |  | **(% GLP-1RA or SGLT2i)** | | | |
| **ASCVD group** | 2012-2015 | 12682 (4.09%) | 1828 (4.57%) | | 310 (4.40%) |
|  | 2016-2018 | 22988 (9.78%) | 5496 (10.5%) | | 832 (9.4%) |
|  | 2019-2021 | 40372 (17.26%) | 8612 (17.46%) | | 1387 (15.41%) |
| **HF group** | 2012-2015 | 2651 (3.16%) | 384 (3.29%) | | 66 (3.15%) |
|  | 2016-2018 | 5429 (7.69%) | 1624 (9.02%) | | 262 (8.47%) |
|  | 2019-2021 | 11199 (15.44%) | 2732 (16.05%) | | 411 (13.32%) |
| **CKD group** | 2012-2015 | 2403 (3.93%) | 307 (4.14%) | | 46 (3.84%) |
|  | 2016-2018 | 6081 (8.47%) | 1395 (9.07%) | | 229 (8.64%) |
|  | 2019-2021 | 12464 (15.99%) | 2640 (16.43%) | | 438 (15.01%) |
| **No ASCVD, HF, or CKD group** | 2012-2015 | 35198 (6.46%) | 5111 (6.93%) | | 849 (6.13%) |
|  | 2016-2018 | 49029 (13.54%) | 9325 (13.38%) | | 1565 (12.40%) |
|  | 2019-2021 | 68232 (20.09%) | 12742 (20.10%) | | 2305 (19.09%) |
| **Total population** | 2012-2015 | 49605 (5.56%) | 7178 (6.05%) | | 1194 (5.48%) |
|  | 2016-2018 | 75637 (11.89%) | 15704 (12.02%) | | 2544 (11.04%) |
|  | 2019-2021 | 115389 (18.81%) | 22811 (18.81%) | | 3944 (17.37%) |
|  |  |  |  | |  |
|  |  | **(% GLP-1RA)** | | | |
| **ASCVD group** | 2012-2015 | 11116 (3.58%) | | 1586 (3.97%) | 273 (3.87%) |
|  | 2016-2018 | 13608 (5.79%) | | 3219(6.15%) | 484 (5.47%) |
|  | 2019-2021 | 25388 (10.86%) | | 5558 (11.27%) | 915 (10.17%) |
| **HF group** | 2012-2015 | 2382 (2.84%) | | 348 (2.98%) | 58 (2.77%) |
|  | 2016-2018 | 3583 (5.07%) | | 1049 (5.83%) | 164 (5.3%) |
|  | 2019-2021 | 7235 (9.98%) | | 1772 (10.41%) | 271 (8.78%) |
| **CKD group** | 2012-2015 | 2224 (3.63%) | | 281 (3.79%) | 42 (3.5%) |
|  | 2016-2018 | 4452 (6.20%) | | 1014 (6.59%) | 153 (5.77%) |
|  | 2019-2021 | 9153 (11.75%) | | 1963 (12.21%) | 336 (11.51%) |
| **No ASCVD, HF, or CKD group** | 2012-2015 | 29954 (5.50%) | | 4343 (5.89%) | 721 (5.20%) |
|  | 2016-2018 | 26160 (7.23%) | | 5042 (7.24%) | 831 (6.59%) |
|  | 2019-2021 | 42326 (12.46%) | | 8259 (13.03%) | 1496 (12.39%) |
| **Total population** | 2012-2015 | 42646 (4.78%) | | 6148 (5.18%) | 1025 (4.70%) |
|  | 2016-2018 | 42268 (6.65%) | | 8864 (6.78%) | 1417 (6.15%) |
|  | 2019-2021 | 72567 (11.83%) | | 14857 (12.25%) | 2598 (11.44%) |
|  |  | **(% SGLT2i)** | | | |
| **ASCVD group** | 2012-2015 | 1994 (0.64%) | | 304 (0.76%) | 44 (0.62%) |
|  | 2016-2018 | 12388 (5.27%) | | 2912 (5.57%) | 444 (5.02%) |
|  | 2019-2021 | 21669 (9.27%) | | 4433 (8.99%) | 684 (7.60%) |
| **HF group** | 2012-2015 | 338 (0.40%) | | 50 (0.43%) | 9 (0.43%) |
|  | 2016-2018 | 2467 (3.49%) | | 729 (4.05%) | 123 (3.97%) |
|  | 2019-2021 | 5606 (7.73%) | | 1354 (7.95%) | 192 (6.22%) |
| **CKD group** | 2012-2015 | 249 (0.41%) | | 37 (0.50%) | 6 (0.50%) |
|  | 2016-2018 | 2212 (3.08%) | | 496 (3.23%) | 92 (3.47%) |
|  | 2019-2021 | 5003 (6.42%) | | 1020 (6.35%) | 149 (5.10%) |
| **No ASCVD, HF, or CKD group** | 2012-2015 | 6605 (1.21%) | | 949 (1.29%) | 152 (1.10%) |
|  | 2016-2018 | 29708 (8.21%) | | 5433 (7.80%) | 915 (7.25%) |
|  | 2019-2021 | 37129 (10.93%) | | 6589 (10.39%) | 1187 (9.83%) |
| **Total population** | 2012-2015 | 8800 (0.99%) | | 1283 (1.08%) | 201 (0.92%) |
|  | 2016-2018 | 43592 (6.85%) | | 8701 (6.66%) | 1417 (6.15%) |
|  | 2019-2021 | 61692 (10.06%) | | 11641 (9.60%) | 1963 (8.65%) |

# Table S11. Factors associated with GLP1RA or SGLT2i therapy among adults with type 2 diabetes living in rural and urban areas of the U.S.

Logistic regression models examined the association between GLP1RA or SGLT2i fills and time-period, rurality, age group, sex, and comorbidity status overall and by index year grouping and were adjusted for time-period, age, sex, rurality, and cardiovascular and kidney comorbidity as appropriate. ASCVD=atherosclerotic cardiovascular disease; CKD=chronic kidney disease; HF=heart failure.

|  | **2012-2015**  OR  (95% CI); P-Value | **2016-2018** OR  (95% CI); P-Value | **2019-2021** OR  (95% CI); P-Value | **Overall** OR  (95% CI); P-Value |
| --- | --- | --- | --- | --- |
| **Time period  (vs. 2012-2015)** |  | | | |
|  | (reference) | 2.49  (2.47-2.52); <0.001 | 4.35  (4.30-4.39); <0.001 | Not applicable |
| **Age**  **(vs. 18-44 years)** |  | | | |
| 45-64 years | 0.84  (0.82-0.86); <0.001 | 0.87  (0.85-0.89); <0.001 | 0.92  (0.90-0.94); <0.001 | 0.88  (0.87-0.89); <0.001 |
| 65-74 years | 0.38  (0.37-0.39); <0.001 | 0.46  (0.45-0.47); <0.001 | 0.54  (0.53-0.56); <0.001 | 0.48  (0.47-0.48); <0.001 |
| > 75 years | 0.11  (0.10-0.11); <0.001 | 0.17  (0.16-0.17); <0.001 | 0.21  (0.20-0.21); <0.001 | 0.17  (0.17-0.18); <0.001 |
| **Sex** |  | | | |
| Male vs. Female | 0.78  (0.77-0.80); <0.001 | 0.95  (0.94-0.96); <0.001 | 0.95  (0.94-0.96); <0.001 | 0.91  (0.90-0.92); <0.001 |
| **Rurality**  **(versus Cities)** |  | | | |
| Small Town Rural | 1.08  (1.05-1.10); <0.001 | 1.06  (1.04-1.08); <0.001 | 1.02 (1.01-1.04); 0.003 | 1.05  (1.04-1.06); <0.001 |
| Remote Rural | 0.99  (0.94-1.06); 0.87 | 0.98  (0.94-1.02); 0.34 | 0.97  (0.93-1.00); 0.062 | 0.98  (0.96-1.00); 0.09 |
| **Comorbidities** |  | | | |
| ASCVD  vs. without ASCVD | 0.91  (0.86-0.96); <0.001 | 1.01  (0.98-1.05); 0.50 | 1.06  (1.03-1.09); <0.001 | 1.03  (1.006-1.05); 0.01 |
| HF  vs. without HF | 0.80  (0.77-0.84); <0.001 | 0.80  (0.78-0.82); <0.001 | 0.92 (0.90-0.94); <0.001 | 0.86  (0.85-0.88); <0.001 |
| CKD  vs. without CKD | 1.14  (1.08-1.19); <0.001 | 1.04  (1.01-1.07); 0.017 | 1.13  (1.10-1.16); <0.001 | 1.12  (1.10-1.14); <0.001 |
| No ASCVD, CKD, or HF vs. ASCVD, CKD, or HF | 0.94  (0.88-0.99); 0.03 | 0.98  (0.94-1.02); 0.32 | 0.95  (0.92-0.98); 0.001 | 0.98  (0.96-0.99); 0.04 |

# Table S12. Characteristics of patients after excluding patients without insurance information available (n=119,964 excluded).

|  | Remote (N=63,725) | Small town (N=353,356) | City (N=2,042,532) | Total (N=2,459,613) | p value |
| --- | --- | --- | --- | --- | --- |
| **Age, mean (SD)** | 65.4 (11.6) | 64.7 (12.0) | 63.9 (12.6) | 64.0 (12.5) | <0.0001 |
| **Age groups, years** |  |  |  |  | <0.0001 |
| 18-44 | 3459 (5.4%) | 22817 (6.5%) | 167541 (8.2%) | 193817 (7.9%) |  |
| 45-64 | 22771 (35.7%) | 129566 (36.7%) | 760955 (37.3%) | 913292 (37.1%) |  |
| 65-74 | 23181 (36.4%) | 125377 (35.5%) | 688930 (33.7%) | 837488 (34.0%) |  |
| >=75 | 14314 (22.5%) | 75596 (21.4%) | 425106 (20.8%) | 515016 (20.9%) |  |
| **Sex** |  |  |  |  | <0.0001 |
| Female | 30453 (47.8%) | 179609 (50.8%) | 1010445 (49.5%) | 1220507 (49.6%) |  |
| Male | 33272 (52.2%) | 173747 (49.2%) | 1032087 (50.5%) | 1239106 (50.4%) |  |
| **Insurance subtype** |  |  |  |  | <0.0001 |
| Commercial | 20034 (31.4%) | 112218 (31.8%) | 808203 (39.6%) | 940455 (38.2%) |  |
| Medicare Advantage | 43691 (68.6%) | 241138 (68.2%) | 1234329 (60.4%) | 1519158 (61.8%) |  |
| **Baseline comorbidity status** |  |  |  |  |  |
| ASCVD | 23688 (37.2%) | 136310 (38.6%) | 751709 (36.8%) | 911707 (37.1%) |  |
| CKD | 6424 (10.1%) | 37518 (10.6%) | 204476 (10.0%) | 248418 (10.1%) |  |
| HF | 7945 (12.5%) | 45297 (12.8%) | 220659 (10.8%) | 273901 (11.1%) | <0.0001 |
| No ASCVD, CKD, HF | 36162 (56.7%) | 195747 (55.4%) | 1179190 (57.7%) | 1411099 (57.4%) |  |
| **Baseline comorbidities** |  |  |  |  |  |
| Atrial fibrillation | 6681 (10.5%) | 33755 (9.6%) | 176566 (8.6%) | 217002 (8.8%) | <0.0001 |
| Amputation | 769 (1.2%) | 4334 (1.2%) | 18976 (0.9%) | 24079 (1.0%) | <0.0001 |
| Cerebrovascular disease | 7906 (12.4%) | 46624 (13.2%) | 258990 (12.7%) | 313520 (12.7%) | <0.0001 |
| Coronary artery disease | 16279 (25.5%) | 91418 (25.9%) | 472721 (23.1%) | 580418 (23.6%) | <0.0001 |
| Hypertension | 54400 (85.4%) | 307261 (87.0%) | 1703787 (83.4%) | 2065448 (84.0%) | <0.0001 |
| Hyperglycemic crisis | 265 (0.4%) | 1831 (0.5%) | 10978 (0.5%) | 13074 (0.5%) | 0.0001 |
| Hypoglycemic crisis | 493 (0.8%) | 3307 (0.9%) | 15636 (0.8%) | 19436 (0.8%) | <0.0001 |
| Neuropathy | 15445 (24.2%) | 92290 (26.1%) | 504106 (24.7%) | 611841 (24.9%) | <0.0001 |
| Other lower extremity complications | 2917 (4.6%) | 16102 (4.6%) | 89286 (4.4%) | 108305 (4.4%) | <0.0001 |
| Peripheral vascular disease | 8708 (13.7%) | 53651 (15.2%) | 328943 (16.1%) | 391302 (15.9%) | <0.0001 |
| Retinopathy | 7888 (12.4%) | 46212 (13.1%) | 283773 (13.9%) | 337873 (13.7%) | <0.0001 |
| Smoking | 8587 (13.5%) | 48789 (13.8%) | 215171 (10.5%) | 272547 (11.1%) | <0.0001 |
| Lower extremity ulcer | 2668 (4.2%) | 14710 (4.2%) | 81963 (4.0%) | 99341 (4.0%) | <0.0001 |
| Revascularization | 5936 (9.3%) | 31408 (8.9%) | 151136 (7.4%) | 188480 (7.7%) | <0.0001 |

# Table S13. Factors associated with GLP1RA or SGLT2i therapy among adults with type 2 diabetes living in rural and urban areas of the U.S. with the addition of insurance type as a covariate.

Logistic regression models examined the association between GLP1RA or SGLT2i fills and time-period, rurality, age group, sex, and comorbidity status overall and by index year grouping and were adjusted for time-period, age, sex, rurality, cardiovascular and kidney comorbidity, and insurance type as appropriate; of note, 119,964 patients were excluded from this analysis due to unknown insurance type. ASCVD=atherosclerotic cardiovascular disease; CKD=chronic kidney disease; HF=heart failure.

|  | **2012-2015**  OR  (95% CI); P-Value | **2016-2018** OR  (95% CI); P-Value | **2019-2021** OR  (95% CI); P-Value | **Overall** OR  (95% CI); P-Value |  |  |
| --- | --- | --- | --- | --- | --- | --- |
| **Time period  (vs. 2012-2015)** |  | | | |  |  |
|  | (reference) | 2.60 (2.57-2.63); <0.001 | 4.57 (4.52-4.62); <0.001 | Not applicable |  |  |
| **Age**  **(vs. 18-44 years)** |  | | | |  |  |
| 45-64 years | 0.87 (0.84-0.89); <0.001 | 0.92 (0.89-0.94); <0.001 | 0.95 (0.92-0.97); <0.001 | 0.92 (0.90-0.93); <0.001 |  |  |
| 65-74 years | 0.59 (0.57-0.61); <0.001 | 0.62 (0.60-0.64); <0.001 | 0.63 (0.62-0.65); <0.001 | 0.62 (0.61-0.63); <0.001 |  |  |
| > 75 years | 0.18 (0.17-0.19); <0.001 | 0.22 (0.22-0.23); <0.001 | 0.24 (0.23-0.25); <0.001 | 0.22 (0.22-0.23); <0.001 |  |  |
| **Sex** |  | | | |  |  |
| Male vs. Female | 0.76 (0.75-0.77); <0.001 | 0.92 (0.91-0.94); <0.001 | 0.93 (0.92-0.95); <0.001 | 0.89 (0.88-0.90); <0.001 |  |  |
| **Rurality**  **(versus Cities)** |  | | | |  |  |
| Small Town Rural | 1.08 (1.05-1.11); <0.001 | 1.10 (1.08-1.12); <0.001 | 1.05 (1.03-1.07); <0.001 | 1.08 (1.07-1.09); <0.001 |  |  |
| Remote Rural | 1.01 (0.95-1.07); 0.87 | 1.00 (0.96-1.05); 0.98 | 1.01 (0.97-1.04); 0.76 | 1.01 (0.98-1.04); 0.46 |  |  |
| **Comorbidities** |  | | | |  |  |
| ASCVD  vs. without ASCVD | 0.95 (0.89-1.00); 0.063 | 1.03 (0.99-1.07); 0.16 | 1.07 (1.04-1.10); <0.001 | 1.04 (1.02-1.06); <0.001 |  |  |
| HF  vs. without HF | 0.86 (0.83-0.90); <0.001 | 0.83 (0.81-0.86); <0.001 | 0.93 (0.91-0.95); <0.001 | 0.89 (0.88-0.91); <0.001 |  |  |
| CKD  vs. without CKD | 1.18 (1.12-1.24); <0.001 | 1.05 (1.02-1.09); <0.001 | 1.13 (1.11-1.16); <0.001 | 1.13 (1.11-1.15); <0.001 |  |  |
| No ASCVD, CKD, or HF vs. ASCVD, CKD, or HF | 0.91 (0.86-0.97); 0.0019 | 0.94 (0.90-0.98); 0.0019 | 0.93 (0.90-0.96); <0.001 | 0.94 (0.92-0.97); <0.001 |  |  |
| **Insurance Status  (versus commercially insured)** |  |  |  |  |  |  |
| Medicare advantage | 0.55 (0.53-0.56); <0.001 | 0.68 (0.67-0.70); <0.001 | 0.82 (0.81-0.84); <0.001 | 0.71 (0.70-0.72); <0.001 |  |  |

#

# References Cited

1. Value Set Authority Center [database on the Internet]. National Library of Medicine. Available from: <https://vsac.nlm.nih.gov/>.

2. Ginde AA, Blanc PG, Lieberman RM, Camargo CA, Jr. Validation of ICD-9-CM coding algorithm for improved identification of hypoglycemia visits. BMC Endocr Disord. 2008 Apr 1;8:4.

3. McCoy RG, Lipska KJ, Van Houten HK, Shah ND. Development and evaluation of a patient-centered quality indicator for the appropriateness of type 2 diabetes management. BMJ Open Diabetes Res Care. 2020 Nov;8(2).

4. Chang HY, Singh S, Mansour O, Baksh S, Alexander GC. Association Between Sodium-Glucose Cotransporter 2 Inhibitors and Lower Extremity Amputation Among Patients With Type 2 Diabetes. JAMA Intern Med. 2018 Sep 1;178(9):1190-8.

5. McEwen LN, Ylitalo KR, Munson M, Herman WH, Wrobel JS. Foot Complications and Mortality: Results from Translating Research Into Action for Diabetes (TRIAD). Journal of the American Podiatric Medical Association. 2016 Jan-Feb;106(1):7-14.
